# Outcome of Cuffed Tunneled Dialysis Catheters for Hemodialysis Patients at a Tertiary Care Hospital: A Descriptive Cross-sectional Study

**DOI:** 10.31729/jnma.4795

**Published:** 2020-06

**Authors:** Kajan Raj Shrestha, Dinesh Gurung, Uttam Krishna Shrestha

**Affiliations:** 1 Department of Cardiothoracic Vascular Surgery, Manmohan Cardiothoracic Vascular and Transplant Center, Institute of Medicine, Maharajgunj, Kathmandu, Nepal.

**Keywords:** *catheter*, *hemodialysis*, *vascular access*

## Abstract

**Introduction:**

Arteriovenous fistula is the most common vascular access for patients requiring hemodialysis, but it is not always possible or practical hence cuffed tunneled dialysis catheter comes into play. The aim of the study was to determine the outcome of cuffed tunneled dialysis catheter used for hemodialysis at a teaching hospital.

**Methods:**

A descriptive cross-sectional study was conducted between January 2014 and December 2019 on 103 chronic dialysis patients with end-stage renal disease presenting to a tertiary care hospital. Ethical approval was received from the institutional review board (2/(6-11) E2/076/77). Whole sampling was done. Data entry and analysis were done in Microsoft Excel 10.

**Results:**

The study included 103 patients with 117 cuffed tunneled dialysis catheters placed for hemodialysis. On assessing the outcome of the catheters, the primary and secondary patency rates of the catheters were 5.85±4.87 and 1.21±3.77 months. Thirty-one (30.1%) patients required one intervention, and 11 (10.68%) catheters required 3 or more interventions to maintain patency. Eighteen (17.48%) patients presented with catheter dysfunction while in 11 (10.68%) cases, the catheter was kinked or malpositioned at the notch. In one patient, procedure was abandoned due to severe bleeding and in 2 (1.94%) patients dialysis catheters could not be negotiated into the right atrium and left in brachiocephalic junction.

**Conclusions:**

Cuffed tunneled dialysis catheter is effective for maintenance hemodialysis in patients with the end-stage renal disease if used with proper care during dialysis even in our setup. The results and outcomes of the procedure are at par with standards.

## INTRODUCTION

Globally, the number of dialysis patients is increasing daily which possesses a challenge concerning permanent vascular access for the hemodialysis.^1^ According to Kidney Diseases Outcome and Quality Initiative (KDOQI), native Arteriovenous fistulas (AVF) are the primary choice of vascular access for long term dialysis as they have excellent patency rates and lower complication rates.^2^

Even then, Cuffed Tunneled Dialysis Catheters (CTDC) are being used by one-third of chronic dialysis population to bridge the vascular access until AVF gets ready or as a permanent route for dialysis.^3^ CTDC were developed more than three decades ago,^4^ and are used due to significant reduction in infectious-complication and long-term usability.^5,6^

Thus, this study aims to determine the outcome of cuffed tunneled dialysis catheters used in End-Stage Renal Disease (ESRD) patients for long term hemodialysis access at a tertiary University Hospital.

## METHODS

This descriptive cross-sectional study was conducted in the Cardiothoracic Vascular Surgery (CTVS) department of Manmohan Cardiothoracic Vascular and Transplant Center (MCVTC) from January 2014 to December 2019. Ethical clearance was taken from the institutional review board for the study (Ref no- 2/ (6-11) E2/076/77). The study included 103 patients with 117 cuffed catheters placed at internal jugular or femoral veins. All patients, planned for the first episode of dialysis (17 patients) and 86 old patients undergoing dialysis were included in the study. All patients in whom the cuffed tunneled dialysis catheter was planned but could not be inserted into the internal jugular vein or femoral vein due to anatomical abnormalities or technical difficulties were excluded from the study.

The catheter used was Surgiwear Permacath 11.5 F and 15 F catheters with a length of 36 cm for right internal jugular vein insertion and 40 cm for the left internal jugular or femoral vein insertion. They were taken to the operating room and vein was punctured percutaneously under ultrasonographic guidance after infiltration of 2% Lignocaine anesthesia 20 ml at the puncture site till the exit site of the catheter along the tunnel. No prophylactic antibiotics were given, as the entire procedure was performed under strict aseptic conditions. The catheter was tunneled from the anterior wall of the axilla to the puncture site subcutaneously and the catheter was inserted under fluoroscopic guidance. The catheters were handled only during dialysis sessions according to the standard protocol of the Dialysis Center. The ports were cleaned with a povidone-iodine solution along with the exit sites before and after the dialysis. During hemodialysis unfractionated heparin 5000 units were given in all cases. At the end of the dialysis, the ports were flushed with heparinized saline. This process was strictly repeated in all cases after dialysis. In the case of catheter thrombosis, Streptokinase 300,000 units were kept in situ for 2-4 hrs or pumped into the catheter via the port. The catheter was removed if it still did not resolve the problem or there was poor flow despite repeated attempts. In case of infection, cultures were obtained through the catheter, peripheral venous blood, and from secretions if any. If the patient presented with signs of infection without other identifiable sources and it did not resolve with a 14-day course of intravenous antibiotics then bacteremia was suspected to be catheter-related and removal of the catheter was advocated. Written Consent was taken from the patients for the procedure. The data were obtained from the hospital record system after appropriate approval from the concerned authorities.

The obtained data was compiled and analyzed in Microsoft Excel 10 worksheet software. Descriptive data were represented as mean±standard deviation. All data were presented as the percentage of total cases done during the study period.

## RESULTS

The study included 103 patients with 117 cuffed tunneled dialysis catheters placed for hemodialysis. On assessing the outcome of the catheters, primary patency duration was 5.85±4.87 months and secondary patency duration was 1.21±3.77 months, hence once the catheter has blocked the chance of the catheter being functional is very less. On follow-up of the catheters, only 96 (82.05%) catheters were patent at 6 months, 72 (61.54%) were patent at 1 year and 59 (50.43%) were patent by 2 years. Thirty (29.13%) patients died due to disease or other issues not related to the catheter while 19 (18.45%) of the patients were lost to follow-up by 2 years.

About 31 (30.1%) catheters required intervention once during the period while 21 (20.39%) required intervention twice and 11 (10.68%) catheters required 3 or more interventions. Approximately 25 (21.37%) catheters had to be explanted due to various reasons.

Regarding complications, in two patients CTDC could not be inserted due to narrow junction between internal jugular and subclavian vein making insertion of larger diameter dilator difficult while in one patient there was excessive bleeding during puncture of a vein leading to hypovolemic shock. Other complications are presented as immediate and long-term complications (Table 1).

**Table 1:** Complications.

Immediate Complications	n (%)
Bleeding leading to shock	1 (0.97)
Infection	3 (2.91)
Arrhythmias	6 (5.83)
Catheter malposition/kinking	11 (10.68)
Technical error	2 (1.94)
Long term complications.	
Catheter thrombus formation	28 (27.18)
Central thrombus formation	13 (12.62)
Central vein stenosis	10 (9.71)
Catheter dysfunction	18 (17.48)

Since the procedure was done under ultrasound guidance and fluoroscopic guidance there was no case of arterial puncture or hemo/pneumothorax. Similarly, all procedures were done in the Trendelenburg position there was also no incidence of air embolism during the procedure.

Among 103 patients, 10 (9.71%) patients had to be catheter had to be replaced twice and in 2 (1.94%) patients it had to be replaced thrice. The male to female ratio was approximately 3:2 (63:40) with male predominance. The age ranged from 22 years to 86 years with a mean age of 67.57± 12.77 years (Table 2).

**Table 2:** Age group distribution.

Age group	n (%)
15-30	3 (2.91)
31-45	10 (9.71)
46-60	20 (19.42)
61-75	51 (49.52)
>76	19 (18.44)
Total	103 (100)

Eighty-six patients in whom catheters were inserted had already undergone hemodialysis and the duration of dialysis was 26.88±24.66 months before insertion of the catheter. There were 17 patients who had not initiated dialysis and were planned for catheter insertion as access for hemodialysis. The most common indication for CTDC was as permanent access either due to the unavailability of the vein or exhaustion of arteriovenous access as shown (Table 3).

**Table 3:** Indication of Cuffed Tunneled Dialysis Catheter (CTDC) insertion.

Indication	n (%)
Maturation of AVF/PD	18 (17.48)
Awaiting living donor transplantation	3 (2.91)
Dialysis bridge following failed previous access and searching for a new one	8 (7.77)
Permanent access	74 (71.84)

The preferred site for catheter insertion is the right internal jugular vein as anatomically it leads directly to superior vena cava and right atrium, and is associated with better patency and fewer complications. Insertion into the left internal jugular vein is associated with an increased incidence of central venous stenosis and poorer patency (Table 4).

**Table 4:** Numbers and site of insertion of catheters.

Site	n (%)
Left Internal Jugular Vein	38 (32.48)
Right Internal Jugular Vein	77 (65.81)
Femoral Vein	2 (1.71)
Total	117 (100)

In our study, 98 (95.15%) patients were hypertensive which was their primary pathology and 54 (52.43%) patients were diabetics (Table 5).

**Table 5:** Comorbidities of the patients.

Comorbidities	n (%)
Hypertension	98 (95.15)
Diabetes Mellitus	54 (52.43)
Heart disease	38 (36.89)
PAD	27 (26.21)
Smoker	20 (19.42)

## DISCUSSION

Cuffed tunneled dialysis catheters are important vascular access adjuncts for hemodialysis and in our study, we successfully inserted 117 catheters over a period of 5 years. Despite this success, we were unable to negotiate the catheters in 3 patients due to anatomical abnormality and bleeding disorder commonly associated with ESRD. Most patients undergoing catheter insertion in our population were elderly (67.57±12.77 years). One reason is that the incidence of ESRD is high in this group, another reason might be they wanted simple procedures for dialysis rather than going for complex ones like preparing native AVF or expensive grafts. Although a native AVF and synthetic grafts are associated with lower complication rates, shorter hospitalization, better outcome, good quality of life and increased patency rates, the place of hemodialysis catheter is irreplaceable.^2^ These catheters are easy to place if done in a controlled environment and the rate of infection is also very low due to presence of cuff, which prevents the bacterial translocation.^6,7^ Native AVF are always the best option but it requires time to prepare and mature and chances of failure are also high. Similarly, synthetic grafts are expensive, difficult to puncture, have higher rates of complications and require frequent interventions compared to catheters.^8^

Concerning the site of insertion of the catheter, according to the guidelines given by KDOQI, the first option is always right internal jugular vein followed by the left internal jugular vein, femoral veins, external jugular veins, subclavian veins and trans lumbar insertion into inferior vena cava.^2^ In this study, there were 2 cases of femoral insertion due to exhaustion of internal jugular veins which led to increased rates of complications.

Ideally, the catheter should be inserted on the opposite side of planned or maturing arteriovenous access. Subclavian veins should be avoided whenever possible because of the risk of venous stenosis and risk of jeopardy to AVF as in our study.^2,8^

The primary and secondary patency of our catheter was similar to studies by Lund et al. and Tesio et al. which quote a wide range of variations ranging between 25% to75% per year.^9,10^ This is probably due to failure in maintaining aseptic environment, general catheter care and proper management of catheter dysfunction and treatment of infection. Catheter design might also have contributed to some of the cases.^11^

The risk of catheter-related bacteremia for tunneled cuffed catheters ranges from 0.016-0.29 per 100 days^12,13^ while catheter infection incidence is between 6-28% in various studies.^4,14,15^ The rate of infection was only 2.91% in our study which is comparatively low. It is difficult to diagnose the catheter-related infection and usually, it is a diagnosis of exclusion as cultures are negative and there is rarely any discharge from the exit site of the catheter to send for culture.

Schwab, et al. reported central venous stenosis in 40% of his patients attributing to subclavian vein stenosis.^4^ The most alluring problem is that this condition recurs despite balloon venoplasty.^16^ In our study, it was seen only in 9.71% of cases. Similarly, 17.48% of our patient had catheter dysfunction which is similar to the studies done by Po CL et al. and Shaffer D who had an incidence of 17-33%.^17,18^

Catheter lumen thrombosis has been as high as 46% and accounts for the majority of catheter dysfunctions which can be treated in 70-90% of cases by injecting fibrinolytic into the catheter.^19^ In our study, it was 27.18% and we used streptokinase for thrombolysis. The disadvantage of streptokinase is that it cannot be reused within 6 months due to the risk of antigenic reaction.

Thrombosis of the central vein is seen in up to 30% of patients with central catheters and is often asymptomatic, although they may present with facial puffiness, arm edema, and decreased flow in the catheter. It can be identified by fluoroscopy or during venogram. Thirteen (12.62%) patients presented with central venous thrombosis, which was partial but limited the flow. In such cases, either an ultra-stiff guidewire was inserted through the catheter or the catheter was withdrawn by a few centimeters. Sometimes, thrombo-suction with a 50ml syringe along with thrombolysis with streptokinase was done to increase the flow. In chronic cases, angioplasty and stenting may be necessary.^20,21^

Catheter kinking or pinching may occur in all procedures of catheter placement. Wong et al. reported this complication in 0.6% of their cases,^22^ while Hamid et al. reported this in 4.9% of cases.^23^ In our study, the rate of catheter kinking or malposition was 10.68%; however, the flow was limited in only a few cases. In order to prevent kinking or malpositioning of the catheter, length should be appropriate and the notch of the catheter should not be acute-angled. Usually, arrhythmia and bleeding during insertion may lead to kinking of the catheter as we might get less time to assess the position of the catheter. Severe kinking may even require its replacement.

Arterial puncture, hemothorax, and pneumothorax are also inadvertent complications encountered during insertion of the catheter but due to the use of ultrasonogram and fluoroscopy, we did not encounter any of these complications as seen in other studies.^24^ Kidney Disease Outcome and Quality Initiative also recommend the use of imaging modalities to insert a catheter to decrease the rate of complication and improve outcome.^2^

## CONCLUSIONS

Our outcomes were at par with other standard institution data. Since the outcome of the catheters that we have inserted was good, cuffed tunneled dialysis catheters can be safe and effective for hemodialysis, especially inpatient with no venous access or with exhausted venous access even in our setup. However, precaution is necessary during insertion and proper care during dialysis is important for the longevity of the catheter. In addition, injudicious use of these catheters may have a deleterious effect in reducing long-term dialysis sites, hence native fistulas should always be first priority.

## Conflict of Interest:

**None.**
